# Universal electronic structure of polar oxide hetero-interfaces

**DOI:** 10.1038/srep14506

**Published:** 2015-09-28

**Authors:** Uwe Treske, Nadine Heming, Martin Knupfer, Bernd Büchner, Emiliano Di Gennaro, Amit Khare, Umberto Scotti Di Uccio, Fabio Miletto Granozio, Stefan Krause, Andreas Koitzsch

**Affiliations:** 1 IFW Dresden, P.O.Box 270116, 01171 Dresden, Germany; 2 Institut für Festkörperphysik, Technische Universität Dresden, 01062 Dresden, Germany; 3 CNR-SPIN, Complesso Universitario Monte S. Angelo, Napoli, Italy; 4 Dipartimento di Fisica, Universita “Federico II” di Napoli, Italy; 5 Department of Physics, Sungkyunkwan University, Suwon, 440-746, South Korea; 6 BESSY II, Albert-Einstein-Str. 15, 12489 Berlin, Germany

## Abstract

The electronic properties of NdGaO3/SrTiO3, LaGaO3/SrTiO3, and LaAlO3/SrTiO3 interfaces, all showing an insulator-to-metal transition as a function of the overlayer-thickness, are addressed in a comparative study based on x-ray absorption, x-ray photoemission and resonant photoemission spectroscopy. The nature of the charge carriers, their concentration and spatial distribution as well as the interface band alignments and the overall interface band diagrams are studied and quantitatively evaluated. The behavior of the three analyzed heterostructures is found to be remarkably similar. The valence band edge of all the three overlayers aligns to that of bulk SrTiO3. The near-interface SrTiO3 layer is affected, at increasing overlayer thickness, by the building-up of a confining potential. This potential bends both the valence and the conduction band downwards. The latter one crossing the Fermi energy in the proximity of the interface and determines the formation of an interfacial band offset growing as a function of thickness. Quite remarkably, but in agreement with previous reports for LaAlO3/SrTiO3, no electric field is detected inside any of the polar overlayers. The essential phenomenology emerging from our findings is discussed on the base of different alternative scenarios regarding the origin of interface carriers and their interaction with an intense photon beam.

Oxide interfaces occasionally show the emergence of novel electronic states that cannot be trivially attributed to any of the constituent materials. A major example of such intriguing behavior is the 2-dimensional electron gas (2DEG) that is formed at the interface between two wide bandgap insulators, i.e. epitaxial LaAlO_3_ and crystalline SrTiO_3_^1^, showing superconductivity and magnetism (possibly coexisting side by side)^2^,^3^,^4^, Rashba-type spin orbit coupling^5^ and a relatively high electron mobility. These findings raise the prospect of stabilizing exotic ground states at properly tailored interfaces and to exploit the electron gas properties in novel all-oxide electronic devices.

Following the seminal work by Ohtomo and Hwang^1^, insulator-to-metal transitions have been found in other heterostructures in which SrTiO_3_ is coupled to a polar band insulator, such as LaGaO_3_, NdGaO_3_^6^,^7^, and NdAlO_3_^8^. LaGaO_3_/SrTiO_3_ and NdGaO_3_/SrTiO_3_ share with LaAlO_3_/SrTiO_3_ the same critical overlayer thickness of *n* = 4 unit cells (u.c.), as well as many features of their transport and spectroscopic properties^6^,^7^,^9^. These findings suggest that the same fundamental mechanisms inducing the formation of the 2DEG take place in all these different structures.

The origin of the metallic state in LaAlO_3_/SrTiO_3_ has been attributed, since its discovery, to the so-called “electronic reconstruction”^1^,^10^. The SrTiO_3_ substrate consists of nominally charge-neutral SrO^0^ and 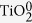 atomic planes, while LaAlO_3_ is ideally composed of alternating charged LaO^+^ and 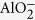 planes, i.e., it bears a large built-in polarization. Associated to this polar state, the electronic reconstruction model foresees an electrostatic field that bends upward the LaAlO_3_ bands away from the interface. When the overlayer exceeds a threshold thickness, the model assumes that the LaAlO_3_ valence band edge is lifted above the SrTiO_3_ conduction band, and that electrons move from the surface of LaAlO_3_ to a SrTiO_3_ interface layer partially screening the polar electric field^11^. The full screening of the field and the complete flattening of the LaAlO_3_ band is only expected to take place for an infinitely thick LaAlO_3_ film. Such complete electronic reconstruction corresponds, in the ionic limit, to the transfer of a charge amounting to 0.5 electrons per in-plane unit cell. Similar concepts are expected to hold for LaGaO_3_/SrTiO_3_ and NdGaO_3_/SrTiO_3_, sharing the same perovskite-related structure and the same nominal cationic valences of LaAlO_3_/SrTiO_3_ both at the A and at the B site.

Several theoretical computations of the LaAlO_3_/SrTiO_3_ electronic structure^12^,^13^,^14^,^15^ confirmed the electronic reconstruction model and suggested that the mobile electrons are confined in a narrow layer close to the interface, within SrTiO_3_, where they populate states with mainly Ti 3*d* character. However, the experimental evidence soon indicated the pivotal role of point defects, e.g. oxygen vacancies, that had not been considered in the early theoretical models. Oxygen vacancies largely affect the transport properties of epitaxial LaAlO_3_/SrTiO_3_ interfaces; furthermore, they can induce insulator-to-metal transition even in amorphous LaAlO_3_, in contrast to the expectations of the electronic reconstruction model^16^. It was hence argued that such defects alone might explain the interface conductivity without resorting to electronic reconstruction. This apparent controversy leaves our present understanding of the origin of the metallic state in polar interfaces at least incomplete to date.

Photoemission spectroscopy (PES) is an unique and versatile probe of the electronic structure of complex systems. It was widely employed to investigate LaAlO_3_/SrTiO_3_^17^,^18^,^19^,^20^,^21^,^22^,^23^, also resorting to resonant photon probes, where the photon energy is tuned to an absorption threshold, e.g. the Ti *L* edge which enhances the Ti 3*d* emission in the valence band by orders of magnitude.

In particular PES was helpful to identify the Ti 3*d* orbital character of the interfacial charge carriers, their concentration and depth profile^18^,^19^,^21^,^24^,^25^. Moreover PES is sensitive to electrostatic fields in the LaAlO_3_ overlayer predicted by the electronic reconstruction scenario by detecting line broadening and shifting^17^,^18^,^19^,^20^,^26^. Such effects are reported to be essentially absent or an order of magnitude smaller than expected. The band bending on the SrTiO_3_ side has been studied in ref. 20,23,27. and the full band alignment across the interface in ref. 20,23. The in-gap states near Fermi energy (*E*_F_) have been characterized by resonant photoemission spectroscopy (ResPES) up to the full *k*-resolved Fermi surface mapping for metallic samples^20^,^22^,^25^,^28^,^29^. Also x-ray absorption spectroscopy (XAS) has been used to study the electronic structure at the LaAlO_3_/SrTiO_3_ interface, in particular the Ti *L* edge and its dichroism^30^,^31^,^32^. The Ti 3*d*_*xy*_ orbital could be thereby identified as the one that is lowest in energy and hosts the 2DEG.

In spite of such broad activity on LaAlO_3_/SrTiO_3_, the similar structures LaGaO_3_/SrTiO_3_ and NdGaO_3_/SrTiO_3_ are almost unexplored^33^. From a methodological point of view it is therefore mandatory to expand the field of investigations to these systems, in order to separate general from material-specific properties and try to identify the electronic features that should be considered as signatures of oxide heterostructures hosting a 2DEG.

In this work, after describing our experiments and sample preparation, we compare LaAlO_3_/SrTiO_3_, LaGaO_3_/SrTiO_3_ and NdGaO_3_/SrTiO_3_ samples with different overlayer thickness in order to investigate in each interface the insulator-to-metal transition, the electronic structure, the concentration and the distribution of interface charges as well as the interface band alignment and the band diagrams. We measured the Ti 2*p* core levels by x-ray photoemission (XPS) and x-ray absorption spectroscopy (XAS) and identified spectroscopic signatures of the 2DEG. These were used to determine the charge concentration and the charge distribution for each heterostructure as a function of the overlayer thickness. By resorting to resonant photoemission spectroscopy (ResPES), we determined the presence and character of interfacial states below the Fermi energy and the alignment of the valence band edges of the materials on both sides of the interface. We hence derived the band diagrams of the three different heterostructures, all sharing the same band bending at the SrTiO_3_ side of the interface and flat valence band edge at the overlayers side. The results are discussed in the light of our present understanding of 2DEGs at oxide interfaces and of possible photo-induced effects. Finally, we conclude the main findings of this paper.

## Experimental

Interfaces of LaAlO_3_/SrTiO_3_, LaGaO_3_/SrTiO_3_, and NdGaO_3_/SrTiO_3_ were fabricated by pulsed laser deposition of the overlayers onto (001)-oriented SrTiO_3_ substrates with an uniform TiO_2_ termination. The growth conditions (730 °C and 1 × 10^−2^ mbar oxygen pressure) and the slow cooling of samples (in the same atmosphere as the growth) were chosen to minimize the formation of oxygen vacancies^34^,^35^, and the Ga desorption. The oscillating intensity of the reflected high-energy electron diffraction (RHEED) pattern, recorded during the growth, allowed to control the overlayers thicknesses and to check the final surface and structural quality. Transmission electron microscope measurements prove the high quality of the investigated samples^6^,^7^,^36^. The synchrotron experiments were carried out at BESSY, beamline UE52-PGM equipped with a Scienta R4000 analyzer. The energy resolution for the photoemission experiments across the Ti *L* absorption edge was 350 meV for the wide valence band scans and 150 meV for the low energy region. XAS has been recorded by measuring the drain current (total electron yield, TEY) and by a separate multiplier detector (partial electron yield, PEY) simultaneously. The energy resolution for XAS was 50 meV. Laboratory based XPS measurements have been done with a SPECS PHOIBOS analyzer and monochromatized Al *K*_*α*_ radiation (*hν* = 1486.6 eV) with an energy resolution of about 400 meV. All measurements were performed at room temperature.

## Experimental Results

### XPS and XAS

**Figure 1:**
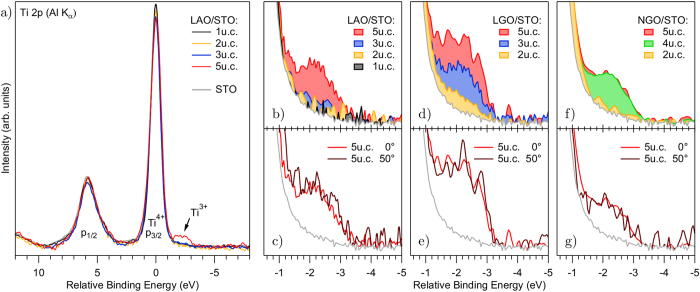
XPS Ti 2*p* spectra of LaAlO_3_/SrTiO_3_, LaGaO_3_/SrTiO_3_ and NdGaO_3_/SrTiO_3_ for different layer numbers. (**a**) Ti 2*p* spectra of LaAlO_3_/SrTiO_3_ and SrTiO_3_ for comparison. The spectra are subtracted by a Shirley background and normalized to the area of the Ti^4+^ 2*p*_3/2_ peak. The energy scale is referenced to the Ti 2*p*_3/2_ peak maximum. The Ti^3+^ states appear as a low energy shoulder of the main Ti^4+^ peak. (**b**–**g**) Ti^3+^ region as a function of layer number and emission angle for the three different materials. For comparison, the gray baseline in all graphs represents the spectrum of a SrTiO_3_ substrate.

In Fig. 1, we show the XPS Ti 2*p* core levels of LaAlO_3_/SrTiO_3_, LaGaO_3_/SrTiO_3_ and NdGaO_3_/SrTiO_3_ samples with overlayer thickness in the range 1–5 u.c. The appearance of a low energy shoulder on the 2*p*_3/2_ line is ascribed to a chemical shift separating the contribution of Ti^4+^ and Ti^3+^ ions. The relative intensity of the Ti^3+^ component increases with increasing overlayer thickness. In agreement with previous studies, a Ti^3+^ component is present well before the threshold of metallicity, i.e. before 4 u.c. thickness^18^,^19^. The 3+ valence state of Ti is directly associated to the occupation of the otherwise empty Ti 3d interface states. This allows the evaluation of the charge carrier density from the ratio between the intensity of the Ti^3+^ and Ti^4+^ components, obtained by a standard fitting procedure. The ratio is in the range 2–4% for 5 u.c. samples, in agreement with previous reports^18^,^25^. These values can be translated into the 2-dimensional electronic charge density *n*_*sheet*_ by considering an effective sampling thickness equal to the inelastic mean free path (IMFP), estimated here ≃2 nm^37^. The results fall in the range 0.8–1.5 × 10^14^ cm^−2^. This should be considered as a lower boundary, because the actual charge distribution may stretch out beyond the IMFP. On the other hand, the estimates may exceed the sheet carrier density as determined by transport measurements, if only a part of the interfacial electrons are mobile. In spite of these concerns, the reported values are in the range of previous XPS estimations for the LaAlO_3_/SrTiO_3_ system^18^,^24^,^25^, and also compare reasonably with Hall measurements^6^,^7^,^8^. No attempt was made to evaluate any systematic material-dependent variation, as the carrier density critically depends on the growth conditions of the single sample. Such dependence may be slightly different from one material to another.

The Fig. 1c,e,g monitor the emission angle dependence of the relative intensity of the Ti^3+^ shoulder for a *n* = 5 u.c. thick sample. The intensity is constant as a function of the angle, within the experimental error, suggesting that the relative Ti^3+^ content is homogeneously distributed perpendicular to the interface at least on a thickness of the order of the IMFP. This is in agreement with previous estimations from HAXPES (high energy photoemission spectroscopy)^19^, but differs from other studies^22^.

**Figure 2:**
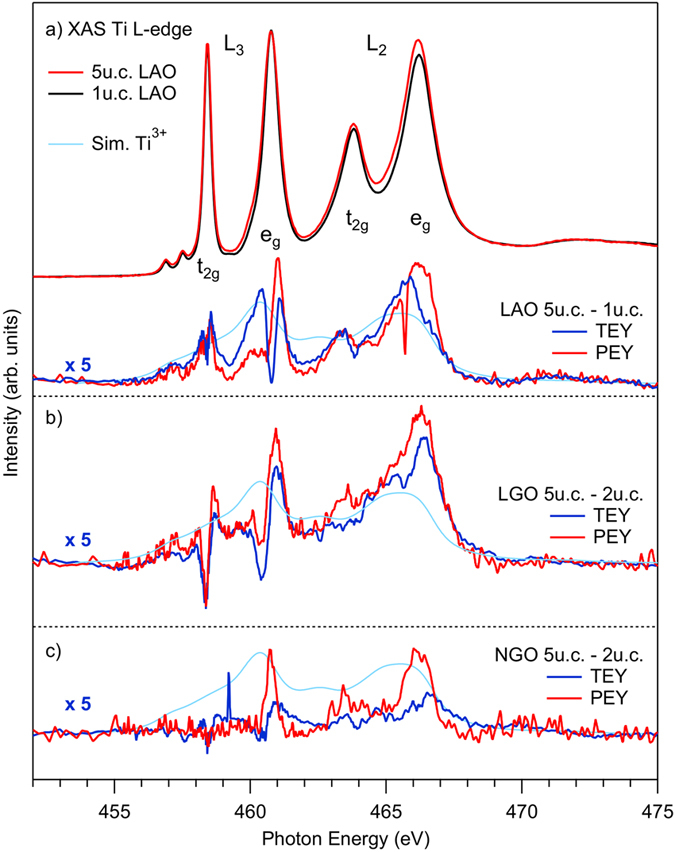
(**a**) upper part: LaAlO_3_/SrTiO_3_ XAS Ti *L* spectra for 5 u.c. and 1 u.c. samples taken in TEY. Difference spectra between thick and thin LaAlO_3_/SrTiO_3_ ((**a**) lower part), LaGaO_3_/SrTiO_3_ (**b**) and NdGaO_3_/SrTiO_3_ (**c**) samples taken in different yield modes and compared to the simulated Ti^3+^ spectra. The vertical axis for the difference spectra is always the same.

Figure 2 shows the XA spectra across the Ti *L* edge. In addition to the spin orbit splitting (*L*_3_, *L*_2_), the crystal field splitting (*t*_2g_, *e*_g_) causes in total the appearance of four lines. The Ti^3+^ signal is also present in the absorption spectra, but distributed over a larger energy range and therefore more difficult to distinguish from the stronger Ti^4+^ signal. Figure 2a (upper part) shows the TEY XAS for 1 u.c. and 5 u.c. thick LaAlO_3_/SrTiO_3_ samples. In order to make the Ti^3+^ part visible we took the difference between these spectra and plot it in the lower part of Fig. 2a, both for PEY and TEY. For comparison a simulated Ti^3+^ spectrum is presented (light blue line)^38^. The match between experiment and model is reasonable. Note, that the sharp oscillations around the original Ti^4+^*L*_3_ lines are due to subtle changes in the peak positions and width as a function of layer number, which are not considered in the simulations.

In PEY, the low energy electrons are filtered out by a retarding potential (*U*_ret_ = 300 V) to tune the depth sensitivity of the detected absorption signal. For the highest values of the retarding potentials employed in this experiment, the PEY probing depth approaches the photoemission inelastic mean free path (IMFP = 1.1 nm at *hν* = 450 eV). In TEY, the probing depth is larger (≃3–4 nm)^39^,^40^. The LaAlO_3_/SrTiO_3_ difference spectra in Fig. 2a are of similar shape and magnitude, indicating that the region of high Ti^3+^ concentration near the interface extends clearly more than 2 nm towards the bulk. This is in agreement with the above discussed depth profiling by XPS measurements. Similar considerations also hold for the differential absorption spectra for 5 u.c. and 2 u.c. samples of LaGaO_3_/SrTiO_3_ (Fig. 2b). For the NdGaO_3_/SrTiO_3_ samples (Fig. 2c) the TEY intensity falls below the PEY intensity indicating that the 2DEG starts to decay around 3–4 nm. We suspect that this difference is related to the overall lower charge concentration of this sample compared to LaAlO_3_/SrTiO_3_ and LaGaO_3_/SrTiO_3_. The difference spectra of LaAlO_3_/SrTiO_3_ and LaGaO_3_/SrTiO_3_ are larger than NdGaO_3_/SrTiO_3_ indicating a larger Ti^3+^ concentration in good accordance with the XPS results.

### ResPES

**Figure 3:**
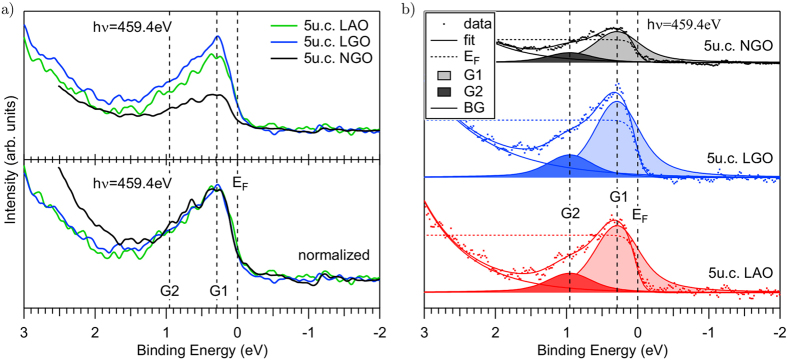
Resonant photoemission at the Ti *L* edge near *E*_F_. (**a**) Comparison of the experimental data. Upper part: Intensity scale normalized to the shallow core levels (Sr 4*p*). (Lower part) Normalization to maximum intensity reveals identical lineshapes. (**b**) Simulation of the spectra, including a smooth background coming from the main valence band, two Voigt peaks (for localized and delocalized states respectively), and the Fermi edge.

**Figure 4:**
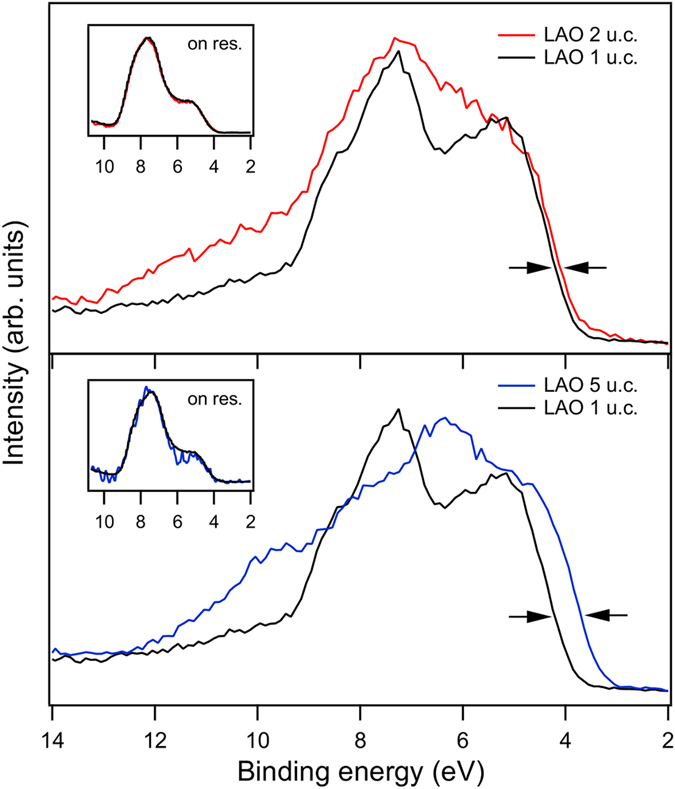
Comparison of valence bands of LaAlO_3_/SrTiO_3_ samples of varying thickness. The energy alignment is carried out using the *L*_3_ resonance enhancement (see insets). The shift of the leading edge for increasing layer thickness corresponds to an upshift of the LaAlO_3_ part (see text for details). The binding energy scale has been set manually to match a valence band maximum of the 1 u.c. LaAlO_3_ of *E*_B_ ≃ 4eV.

The Ti 3*d*^1^ states just below the Fermi energy (*E*_F_) can be efficiently analyzed by ResPES^25^,^28^,^29^,^41^,^42^. Such states have a gap-less excitation spectrum and are therefore responsible for the transport properties of the system; in other words, they host the 2DEG. Figure 3 presents the near-*E*_F_ photoemission signal collected with a photon energy of *hν* = 459.4 eV, close to the energy region where the Ti^3+^ absorption has a maximum. This intensity is actually small and only visible on-resonant. Under this condition the Ti 3*d*^1^ states are strongly enhanced. The intensity upturn towards higher energy in Fig. 3a is due to the presence of the main valence band, shown in Fig. 4, where also resonance effects occur. The spectra are normalized to the shallow core levels (Sr 4*p*, not shown) at higher binding energy (*E*_B_). The magnitude of the Ti 3*d*^1^-related emission is maximal for the LaGaO_3_/SrTiO_3_ sample and minimal for the NdGaO_3_/SrTiO_3_ sample, which qualitatively agrees with the above reported XPS and XAS results. However, the normalization in the lower panel of Fig. 3a reveals that the shape of the spectra is identical. Hence, we conclude that the different magnitudes are not related to different degrees of band filling (that would lead to a different Fermi level) but rather to differences in the density of coherent regions, i.e. to sample inhomogeneities. Note that, similarly to the observation for the core levels, near-*E*_F_ intensity is sometimes also found for samples with *n* < 4 u.c., although they are insulating.

In Fig. 3b the ResPES plots near *E*_F_ are further analyzed. First we ascertain that the energy width of these spectra exceeds 1 eV, which is well below the band bottoms at 0.3 eV binding energy observed by ARPES^29^,^41^,^43^. This suggests that states at higher binding energies are present. The near *E*_F_ peak has an asymmetric lineshape similar to the one observed by Drera *et al.*^23^. The spectra are successfully simulated by two Voigt peaks, G1 and G2, a smooth tail from the main valence band and a cut-off at the Fermi edge. The existence of two components is in agreement with previous studies^25^,^41^, and has been associated with the coexistence of localized defect states (G2) and delocalized bandstates (G1). Their amplitude, however, cannot be directly related to the density of occupied Ti 3*d* states, since the cross section of the resonant photoemission process depends strongly on *hv*^44^.

### Band Alignment

In the previous section we analyzed the spectral features related to the presence of Ti 3*d*^1^ states, a direct signature of the interfacial 2DEG at the interface. In order to achieve a more comprehensive understanding of our heterostructures, a full band diagram across the interface needs to be derived^45^, including the details of interface band bending and of the valence band offset (VBO) between SrTiO_3_ and the overlayers.

The interfacial band alignment at the LaAlO_3_/SrTiO_3_ interface has been investigated before by several authors and by different analytical schemes. The VBO between LaAlO_3_ and SrTiO_3_ can be inferred from a fitting procedure by using the energy and the magnitude of the valence bands of pure LaAlO_3_ and SrTiO_3_ as fitting parameters^20^,^21^. Drera *et al.* use ResPES at the Ti *L* and La *M* edge to deconvolve a given valence band in its contributions and evaluate thereby their offset^23^. Alternatively, the energy difference between core level pairs of the heterostructure can be referenced to the constituting materials^19^,^46^. The values for the VBO obtained in these ways vary to a certain degree in the literature. Positive values were obtained by Qiao *et al.* (0.16 eV for 3 u.c.)^47^ and Berner *et al.* (0.3–0.4 eV for 4 u.c.)^20^. The positive sign corresponds to the situation where the LaAlO_3_ valence band maximum (VBM) is closer to *E*_F_ than SrTiO_3_. This is referred to as type II interface. Negative values are reported by Segal *et al.* (−0.35 eV for 4 u.c.)^17^ and Drera *et al.* (−0.1 eV for 5 u.c.)^23^ (type I interface). Chambers *et al.* showed a sample and preparation dependence for *n* = 4 u.c. in the range of −0.06 … +0.34 eV.

In the following we derive interfacial energy offsets by evaluating ResPES data and core level energy differences. Figure 4 contains ResPES data of the main valence band. As mentioned above the samples show moderate thickness dependent charging effects. Therefore, the first step is to find an energy reference for samples with different thicknesses. The shallow core levels around *E*_B_ = 20 eV cannot be used for this purpose because their shape changes drastically depending on *n*. Therefore, we aligned the spectra by their resonance enhancements, defined as the difference between on-resonant and off-resonant emission at the Ti *L* edge.

These resonant enhancements do not depend on *n* in the main valence band region and can be used to accurately align the SrTiO_3_ related on- and off-resonant emission. It is seen in Fig. 4 that the leading edge of the 5 u.c. sample shifts by appr. Δ*E* = 0.5 eV towards lower energies compared to the 1 u.c. sample. Since the SrTiO_3_ part of the valence band is fixed by the alignment, the leading edge must originate from the LaAlO_3_ part of the valence band. From the difference the energy shift Δ*E* is obtained. The resonance enhancement occurs at the *L*_3_ and the *L*_2_ edge likewise. We measured both and took the average Δ*E*.

**Figure 5:**
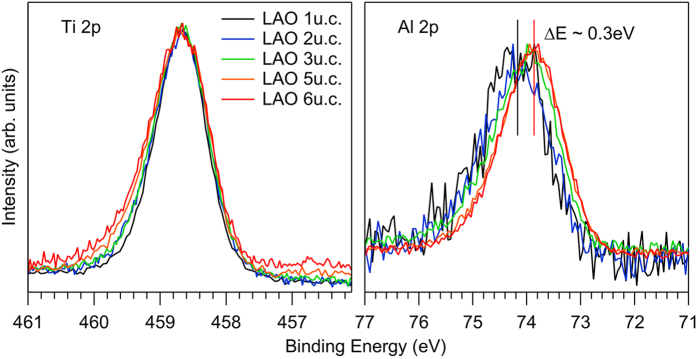
Ti 2*p* and Al 2*p* XPS core levels from LaAlO_3_/SrTiO_3_ samples with varying thickness aligned to the Ti 2*p* peak position. The Al 2*p* peak shifts to lower energies with increasing layer number.

**Figure 6:**
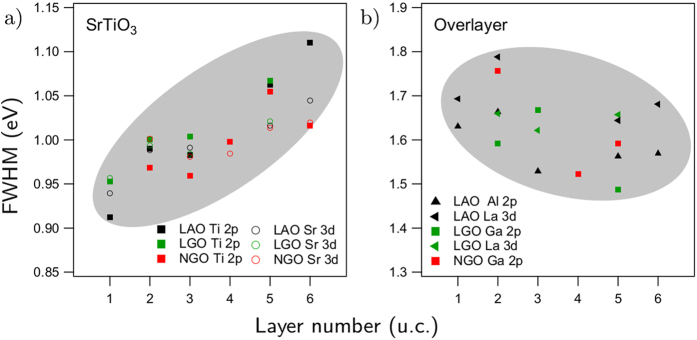
Full width half maximum (FWHM) of the specified core levels as a function of layer number. (**a**) SrTiO_3_ related lines. The energy axes refers to the FWHM of Ti 2*p*_3/2_. The FWHM of Sr 3*d*_5/2_ is shown with an offset of +0.09 eV to enable convenient comparison. (**b**) overlayer related lines. The energy axes refers to Ga 2*p*_3/2_, the offsets for Al 2*p* is +0.5 eV and for La 3*d*_5/2_ −0.2 eV. The gray contours are guides to the eye and emphasize the positive/negative slope of the data points.

The same thickness dependent energy shifts must also occur for the core levels because their energy position is rigidly connected to the valence band. Figure 5 collects the Ti 2*p* and Al 2*p* XPS core levels as a function of *n* for LaAlO_3_/SrTiO_3_. Now the relative energy scale is set by aligning them to the Ti 2*p* peak maximum. Indeed the energy shift of the LaAlO_3_ related Al 2*p* emission to lower energies is apparent in accordance with the observation from the valence band. The energy shifts relative to the 1 u.c. sample are obtained by standard fitting procedures (not shown). In the case of LaGaO_3_/SrTiO_3_ and NdGaO_3_/SrTiO_3_, the Ga 2*p* line has been used instead of the Al 2*p*. Moreover, it is observed in Fig. 5 that the width of the Ti 2*p* line increases with *n*. In fact, this is a general phenomenon for all SrTiO_3_ related lines, which is absent for the overlayer. Figure 6 compiles the full width half maximum (FWHM) for several SrTiO_3_ and overlayer related core levels. Clearly, the SrTiO_3_ lines show an increasing linewidth whereas the opposite is found for the overlayer (gray contours emphasize the positive/negative slope of the data points).

**Figure 7:**
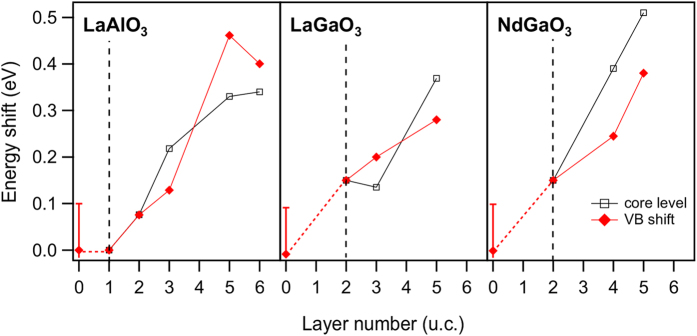
Energy shifts obtained from core level and valence band evaluation. The dashed vertical line highlights the reference sample thickness from where the core level shifts are counted. *n* = 0 corresponds to the SrTiO_3_ substrate. A free surface is involved for this data point which entails increased error bars.

Figure 7 collects the above described energy shifts for all measured LaAlO_3_, LaGaO_3_ and NdGaO_3_ samples for the valence band referenced to SrTiO_3_ and for the core levels relative to the 1(2) u.c. samples. We find reasonable agreement between valence band and core level energy shifts, both extracted by experimentally independent procedures.

Now we discuss the origin of the observed energy shift Δ*E* between substrate and overlayer. Note, that we used relative energies so far. On an absolute energy scale the observed energy shift could appear due to an upshift of the LaAlO_3_ levels or a downshift of the SrTiO_3_ levels, respectively. An upshift of the LaAlO_3_ levels would occur when an electrostatic potential inside the overlayer is present and increases with *n*, i.e. the polar catastrophe scenario. A downshift of the SrTiO_3_ levels would be realized in case of a successive band bending at the SrTiO_3_ side. Both scenarios affect the peakshape of the SrTiO_3_ and the overlayer differently: in the former case significant peak broadening effects would be expected for the overlayer, in the latter case for the substrate. Based on Fig. 6, we can therefore rule out the presence of strong electric fields in the overlayer and argue for a near interface SrTiO_3_ band bending of appr. 0.4 eV magnitude. For the 6 u.c. LaAlO_3_/SrTiO_3_ sample saturation effects are seen, i.e. the 2DEG has fully formed by this time.

For the construction of the band diagram across the interface, knowledge of the interfacial potential well at the SrTiO_3_ side is not sufficient. An important quantity in this respect is the intrinsic VBO for a sample of given thickness, i.e. the alignment of the VBM far from the interface. This is generally difficult to distinguish from the near interface region by spectroscopic means and we suspect that this difficulty has contributed to the diverging values of the VBO mentioned above. Here we notice that the energy shifts Δ*E* in Fig. 7 extrapolate to zero for *n* → 0, i.e. zero band bending. This would not be the case for a finite intrinsic VBO, e.g. for a VBO ≫ 0 the VBM of LaAlO_3_ and SrTiO_3_ would always substantially differ. Therefore, we determine the intrinsic VBO to be close to zero. An error of ±0.15 eV is estimated from the scattering of the data points in Fig. 7.

**Figure 8:**
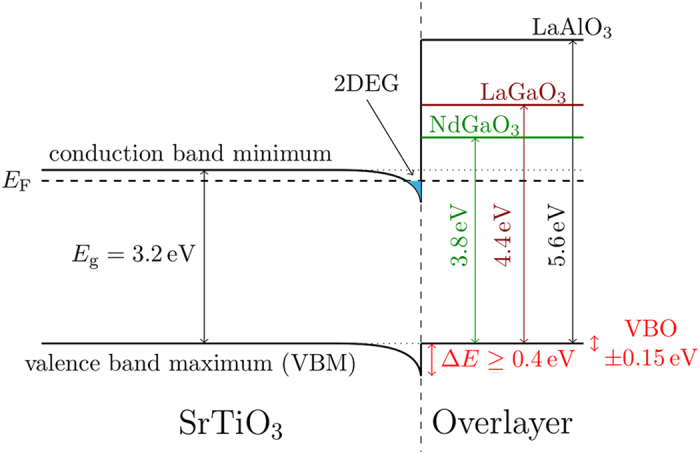
Band diagram of polar oxide heterostructures derived from photoemission data.

The quantitative information about the electronic structure of our samples reported in Figs 4, 5, 6, 7 is employed to sketch the band diagram shown in Fig. 8 for separately describing the three different hetero-interfaces with *n* = 5 u.c. We briefly comment on the main features: a) The diagram in Fig. 8 shows a bending of the valence band in SrTiO_3_ close to the interface region. Its depth is of the order of 0.4 eV (as estimated from data in Fig. 7). This should be considered as a lower limit, as the experimental energy shifts are affected by the integration of the signal over a finite probing depth. The extension of this region is estimated ≥2 nm by XPS performed at different emission angles. b) The bands of the overlayer are flat. In other terms, we do not observe any built-in electric field in this region. c) The valence band edge of the overlayer is aligned to the valence band edge of bulk SrTiO_3_. d) The three hetero-interfaces show different conduction band offsets as a consequence of the valence band alignment and of the difference in the gaps of the three considered overlayers.

## Discussion

The band diagram in Fig. 8 confirms and quantifies the presence of an interface band bending and, as a consequence, the formation of a confining potential at the SrTiO_3_ side of the interface. More importantly, it highlights the major similarities in the interface electronic structure of the three different investigated systems, thus identifying the crucial electronics signatures of 2D electron gases in oxide heterostructures.

As mentioned above, previously published results on the interfacial band alignment in LaAlO_3_/SrTiO_3_ are quite scattered. This concerns the sign and magnitude of the VBO^17^,^20^,^21^,^46^,^47^ as well as presence and depth of the SrTiO_3_ potential well^23^,^24^,^27^,^46^. By considering the *n*-dependence of the energy shift and the FWHM of substrate and overlayer related core levels respectively the band bending at the SrTiO_3_ side is clearly observed in qualitative agreement with^23^,^27^. Our energy shift of Δ*E* ≥ 0.4 eV is consistent with ref. 23. (Δ*E* = 0.7 eV) obtained by a similar ResPES method. Our study extends the results to the case of LaGaO_3_/SrTiO_3_ and NdGaO_3_/SrTiO_3_, showing that the three systems share a very similar response in terms of SrTiO_3_ valence band bending. We show that the VBO in the limit of zero overlayer thickness is negligible in all the hetero-interfaces within an error estimated in the order of 0.15 eV. The SrTiO_3_ valence band bending implies a similar bending of the conduction band, which crosses *E*_F_ and hosts the 2DEG. The bandwidth of these states depends on the optical gap of SrTiO_3_ and the position of the VBM relative to *E*_F_. With *E*_g_ = 3.2 eV and VBM = 3.4 eV we obtain a value of ≥0.2 eV consistent with the conduction band spectra in Fig. 3, where the maximum emission is located at *E*_B_ = 0.3 eV. This value corresponds to the conduction band bottom because the density of states must have a maximum there. The interface band bending and the formation of an interfacial potential well are assumed to be directly related to the properties of the second harmonic generation in LaAlO_3_/SrTiO_3_^9^,^48^.

The right side of the band diagram in Fig. 8 shows flat bands in the overlayer region, as proved by the absence of any significant line broadening in the core levels of the relevant cations and of the valence band edge at the overlayer side. Similar observations are reported in a number of previous experiments^17^,^18^,^19^,^20^,^21^,^23^,^46^. Our data show that such band flatness is a general feature of PES measurements on oxide heterostructures hosting a 2DEG. We now discuss how far the band diagram in Fig. 8 is in agreement with a number of different proposed scenarios explaining the 2DEG formation. A very straightforward analysis, based on the one-dimensional Poisson equation, associates the negative value of the second derivative of the electronic potential at the SrTiO_3_ side of the interface to the presence of a net negative charge. This feature is hard to reconcile with a model assuming that both the electrons and the donors occupy the same region within SrTiO_3_ because this would lead to local charge neutrality. Instead, it is compatible with any model assuming donor states either a) located exactly at the interface, or b) embedded in the overlayer or c) located at the external surface of the overlayer, as foreseen in the electronic reconstruction model. The scenarios mentioned above result in different predictions for the electric field in the overlayer. While the presence of positively charged donor state at the interface would be compatible with a flat potential in the overlayer, the electronic reconstruction model foresees a built-in electric field of ≃1V/u.c. Our measurements, as well as previously published ones performed on LaAlO_3_/SrTiO_3_, failed to observe such field. Incidentally, an electric field was instead observed in XPS measurements in the overlayer of a heterostructure that does not host a mobile electron gas, LaCrO_3_/ SrTiO_3_, based on the small bandgap polar Mott insulator LaCrO_3_^26^. One possible straightforward explanation of our results is that no polar field is present in LaAlO_3_/SrTiO_3_, LaGaO_3_/SrTiO_3_ and NdGaO_3_/SrTiO_3_ and that conductivity is due, e.g., to defects induced into SrTiO_3_ by the fabrication process. Such scenario has been convincingly invoked to explain conductivity in SrTiO_3_ single crystals covered by an amorphous overlayer^16^. This hypothesis, suggesting a complete breakdown of the electronic reconstruction scenario, is anyway at odds with a number of experiments that confirmed the presence of a strong electric polarization of the LaAlO_3_ lattice^36^, and directly measuring the presence of an electrical potential gradient in the LaAlO_3_ layer^49^,^50^, although smaller than predicted^12^,^13^,^14^.

A revised version of the electronic reconstruction model, including the effect of point defects in the overlayer, was recently proposed^51^,^52^,^53^. Just as in the classic electronic reconstruction model, the polar catastrophe is solved by the transfer of electrons from the polar overlayer into SrTiO_3_. Cation intermixing at the interface, on the other hand, has been studied in ref. 6,7,36. on samples produced in the same system and in similar conditions, highlighting the presence of sharp interfaces. However, it is proposed that the electronic reconstruction is mediated by the formation of surface oxygen vacancies in LaAlO_3_. The correspondent surface in-gap states then would act as donor states for the 2DEG. Unlike the standard electronic reconstruction scenario, for which a residual field is expected in LaAlO_3_ for any finite thickness, this oxygen-vacancy-mediated mechanism could allow in principle a complete screening of the polar field. Such oxygen-vacancy-mediated scenario is consistent with the band diagram reported in Fig. 8, including the presence of a net charge transfer from the LaAlO_3_ to the SrTiO_3_ side of the interface. Still, the above described scenario does not solve the inconsistency between the data collected from photon-based spectroscopies, that observe no band bending in LaAlO_3_, and the data collected by other techniques (e.g^36^,^49^,^50^.) that did confirm the presence of a polar field in LaAlO_3_. A mechanism that could explain why the presence of an electric field in LaAlO_3_ is not observed in photon-based spectroscopies, while being observed by other investigation techniques, is related to photon doping.

The residual field expected in LaAlO_3_ after electronic reconstruction for any finite thickness can possibly be screened by an extra “photo-induced electronic reconstruction”, as suggested in ref. 7. In this scenario, the system is assumed to approach under a photon beam a steady-state in which additional light-induced *e*-*h* pairs complete the screening of the ionic polar field. At the same time, a “photo-induced electronic reconstruction” might explain the experimentally observed long lifetime of light-induced *e*-*h* pairs (and the consequent persistent photoconductivity)^54^ and the absence of any broadening in the observed La, Nd, Al, Ga photoemission lines. The presence of obvious photo-induced effects was probed by performing dedicated time- and flux-dependent experiments. No clear evidence for such effects was found. On the contrary, we found qualitative accordance of the Ti^3+^ concentration in different XPS, XAS and ResPES measurements performed by spanning an effective photon flux by two orders of magnitude. Nevertheless, the possible quick saturation of the system under light towards a perturbed and long-lived steady-state (as highlighted by photoconductance measurements^7^,^54^), could make the truly unperturbed state experimentally very hard to access and make therefore the separation of photoinduced from intrinsic properties quite elusive.

## Conclusions

The electronic structure of LaAlO_3_/SrTiO_3_, LaGaO_3_/SrTiO_3_, and NdGaO_3_/SrTiO_3_ heterostructures was investigated by x-ray photoemission spectroscopy, x-ray absorption spectroscopy (in total and partial electron yield) and resonant photoemission spectroscopy. The electronic properties of the three investigated systems were investigated across the insulator-to-metal transition by comparing the spectral response for samples below and above the critical thickness. Our data highlight the presence of major similarities underlying the properties of the three investigated systems. In particular, a bending of the SrTiO_3_ valence band is observed, which gradually builds up for all the materials and reaches Δ*E* ≥ 0.4 eV for *n* = 5 u.c. In the overlayer, on the other hand, no electrostatic fields are observed for any layer number. The valence bands of substrate and overlayer align to each other within VBO ≤ 0.15 eV. The induced charge carriers are always of Ti 3*d* character, have a near interface concentration in the range of 0.8–1.5 × 10^14^ cm^−2^ and extend at least 2 nm towards the substrate.

Our observations are consistent with defect assisted versions of the electronic reconstruction, where surface donor states provide the charge subsequently transferred to the interface. While a direct photon flux dependence of our measurements is not observed, a quick saturation towards a photo-induced metastable state, preventing the observation of truly unperturbed states under the probing photon beam, can not be ruled out.

## Additional Information

**How to cite this article**: Treske, U. *et al.* Universal electronic structure of polar oxide hetero-interfaces. *Sci. Rep.* **5**, 14506; doi: 10.1038/srep14506 (2015).
